# Typhoid fever and paratyphoid fever: Systematic review to estimate global morbidity and mortality for 2010

**DOI:** 10.7189/jogh.02.010401

**Published:** 2012-06

**Authors:** Geoffrey C. Buckle, Christa L. Fischer Walker, Robert E. Black

**Affiliations:** Johns Hopkins University Bloomberg School of Public Health, Baltimore, Maryland, USA

## Abstract

**Background:**

Typhoid and paratyphoid fever remain important causes of morbidity worldwide. Accurate disease burden estimates are needed to guide policy decisions and prevention and control strategies.

**Methods:**

We conducted a systematic literature review of the PubMed and Scopus databases using pre-defined criteria to identify population-based studies with typhoid fever incidence data published between 1980 and 2009. We also abstracted data from annual reports of notifiable diseases in countries with advanced surveillance systems. Typhoid and paratyphoid fever input data were grouped into regions and regional incidence and mortality rates were estimated. Incidence data were extrapolated across regions for those lacking data. Age-specific incidence rates were derived for regions where age-specific data were available. Crude and adjusted estimates of the global typhoid fever burden were calculated.

**Results:**

Twenty-five studies were identified, all of which contained incidence data on typhoid fever and 12 on paratyphoid fever. Five advanced surveillance systems contributed data on typhoid fever; 2 on paratyphoid fever. Regional typhoid fever incidence rates ranged from <0.1/100 000 cases/y in Central and Eastern Europe and Central Asia to 724.6/100 000 cases/y in Sub-Saharan Africa. Regional paratyphoid incidence rates ranged from 0.8/100 000 cases/y in North Africa/Middle East to 77.4/100 000 cases/y in Sub-Saharan Africa and South Asia. The estimated total number of typhoid fever episodes in 2010 was 13.5 million (interquartile range 9.1–17.8 million). The adjusted estimate accounting for the low sensitivity of blood cultures for isolation of the bacteria was 26.9 million (interquartile range 18.3–35.7 million) episodes. These findings are comparable to the most recent analysis of global typhoid fever morbidity, which reported crude and adjusted estimates of 10.8 million and 21.7 million typhoid fever episodes globally in 2000.

**Conclusion:**

Typhoid fever remains a significant health burden, especially in low- and middle-income countries. Despite the availability of more recent data on both enteric fevers, additional research is needed in many regions, particularly Africa, Latin America and other developing countries.

Typhoid and paratyphoid fever remain important public health problems globally and major causes of morbidity in the developing world [1]. Typhoid and paratyphoid fever are acute and often life-threatening febrile illnesses caused by systemic infection with the bacterium *Salmonella enterica* serotype typhi and paratyphi, respectively. Classical symptoms include gradual onset of sustained fever, chills, hepatosplenomegaly and abdominal pain. In some cases, patients experience rash, nausea, anorexia, diarrhea or constipation, headache, relative bradycardia and reduced level of consciousness [2]. While both diseases share clinical features, paratyphoid fever tends to have a more benign course of illness. Without effective treatment, typhoid fever has a case-fatality rate of 10–30%. This number is reduced to 1–4% in those receiving appropriate therapy [1].

The most recent global burden of disease estimates for typhoid and paratyphoid fever reported that in 2000, there were 22 million new cases of typhoid fever, 210 000 typhoid fever-related deaths, and 5.4 million cases of paratyphoid fever [1]. This study offered improved estimates from past updates and analyses [1,3-6].

A revised estimate of the global burden of typhoid and paratyphoid fever is critically needed for developing improved strategies for disease prevention and control. The global epidemiology of these diseases has changed with global population growth and provision of clean water and sanitation systems. Advances in surveillance, improved understanding of the age distribution of the disease, and more recent studies allow for updated estimates of the global burden of typhoid and paratyphoid fever.

## METHODS

### Systematic review and data extraction

We conducted literature searches in PubMed and Scopus databases using combinations of the following search terms: *typhoid; Salmonella typhi; Salmonella paratyphi; incidence; prevalence; mortality; disease burden; surveillance; distribution*. The initial literature search was conducted in January 2009 and was updated on December 31, 2009. We screened study titles and abstracts focused on typhoid and/or enteric fever according to *a priori* inclusion and exclusion criteria. For papers not excluded based on title and abstract, full text articles were obtained and reevaluated for inclusion/exclusion criteria. We sought to include all studies published from 1980–2009 collecting prospective, population-based typhoid fever incidence data with blood culture confirmation of diagnosis from both active and passive surveillance studies. Intervention studies were included, but estimates were based on non-intervention groups only. Studies published in English, Spanish, Italian, Portuguese, or French were included. We excluded studies that did not diagnose typhoid fever by blood culture or that used stool culture for diagnosis. We also excluded case reports, microbiological reports, studies of carriers, and studies whose results did not allow for separation of *S. typhi* and *S. paratyphi* cases. Studies of hospitalized patients were excluded unless differentiation between inpatients and outpatients was clear; however, studies that screened for typhoid fever among individuals presenting with febrile illness at clinics/hospitals were considered separately from studies of hospitalized patients. We only included systematic review papers and excluded all commentaries. We abstracted data from the annual reports of notifiable diseases in countries with advanced surveillance systems.

### Analytic methods

Because of the scarcity of information, input data for typhoid and paratyphoid fever were grouped into the 7 Super Regions as defined by the Global Burden of Disease Project (Super Region 1: Australasia, Southern Latin America, High Income North America, High Income Asia Pacific; Super Region 2: Western Europe, Eastern Europe, Central Europe, Central Asia; Super Region 3: Southern Sub-Saharan Africa, Central Sub-Saharan Africa, West Sub-Saharan Africa, East Sub-Saharan Africa; Super Region 4: Northern Africa/Middle East; Super Region 5: South Asia; Super Region 6: East Asia, South East Asia; Super Region 7: Caribbean, Andean Latin America, Central Latin America, Tropical Latin America, Oceania) [7]. We estimated the incidence using data from all eligible studies conducted within the corresponding Super Region and regional groupings. For any Super Region lacking data on paratyphoid fever, we extrapolated an incidence estimate from the Super Region with the closest typhoid fever incidence estimate.

Typhoid fever incidence rates were grouped with respect to age (ie, children <5 years and persons ≥5 years) for regions where age-specific data were available. The median proportion of typhoid fever cases observed among children <5 years of age was calculated and this figure was used to derive the estimated proportion of cases among those 5 years of age and older. We then calculated age-specific incidence rates and the annual number of typhoid fever episodes within each age strata using the median proportion of typhoid fever cases among each age group and the estimated number of overall typhoid fever episodes across all ages.

To estimate the number of typhoid fever episodes in each Super Region for 2010, we applied the median incidence for each Super Region to the corresponding population estimates. Uncertainty bounds were calculated using interquartile ranges. The total episodes were summed across Super Regions to provide the crude global typhoid fever burden and estimates of uncertainty.

An adjusted estimate of global typhoid fever burden was also calculated to account for the low sensitivity of the blood culture to isolate *S. typhi* or *S. paratyphi*. Similar to previous estimates by Crump et al., an adjustment factor of 2 was chosen based on a conservative estimate of 50% sensitivity [1]. This figure was the lowest reported sensitivity among 3 studies evaluating this culture method for typhoid fever diagnosis [8-10].

We estimated case-fatality rates for typhoid and paratyphoid fever from the published literature and the surveillance system data and applied to incidence rate estimates to calculate mortality rates.

## RESULTS

**Figure 1:**
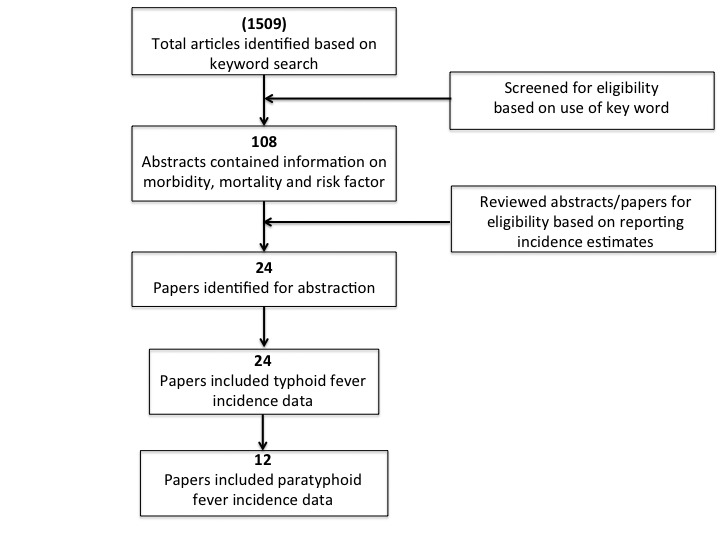
Selection strategy flow diagram used to identify studies on typhoid and paratyphoid fever.

The systematic review yielded 24 studies that examined typhoid fever incidence and employed blood culture as the criteria for diagnosis (**Figure 1**) [11-34]. Five advanced surveillance systems reporting blood-culture confirmed typhoid fever cases were also identified [35-39]. In addition, after the manuscript was accepted, we became aware of one recently published study that met systematic review inclusion criteria, so the analysis was updated to include this data [40]. In total, typhoid fever incidence data was abstracted from 47 countries across 14 (67%) of the 21 regions (**Table 1**). Population-based and prospective vaccine studies contributed data for 13 countries across 8 regions. The remaining incidence data was collected by typhoid fever surveillance systems in the 6 developed regions, each of which includes 1 or more countries with national-level surveillance. The developed regions include: High Income Asia Pacific, High Income North America, Central Europe, Eastern Europe, Western Europe, and Australia/New Zealand. Overall, our analysis includes national-level incidence data from 34 countries across these regions. Paratyphoid fever incidence data was available for 9 countries representing 7 (33%) of the 21 regions (**Table 2**). Only 2 regions included national-level surveillance systems reporting paratyphoid fever incidence (High Income Asia Pacific and Australia/New Zealand). Population-based studies provided paratyphoid fever data for 7 countries in 5 of the regions (Southern Latin America, North Africa/Middle East, South Asia, South East Asia, and East Asia). The median year of data collection for included studies is 2004.

**Table 1:** Input data for typhoid fever incidence rates and summarized median incidence rates by Super Region*

Super Region	Typhoid fever cases	Person-years (p-years)	Incidence (episodes/100 000 p-years)		
**Super Region 1**					
Australia/New Zealand [35,36]	695.0	17 753 491.0	3.9		
	463.0	7 731 880.0	6.0		
Latin America, Southern [11-13]	164.0	136 525.0	120.1		
	68.0	65 718.0	103.5		
	28.0	30 906.0	90.6		
North America, High Income [37]	7503.0	5 250 827 005.0	0.1		
Asia Pacific, High Income [38]	388.0	1 021 033 000.0	0.0		
Europe, Western [39]	31.0	33 211 134.0	0.1		
	207.0	31 918 266.0	0.6		
	10.0	3 131 242.0	0.3		
	50.0	21 861 785.0	0.2		
	35.0	21 159 333.0	0.2		
	832.0	255 015 133.0	0.3		
	468.0	328 973 094.0	0.1		
	38.0	44 771 106.0	0.1		
	51.0	17 372 910.0	0.3		
	219.0	237 547 356.0	0.1		
	1.0	1 922 572.0	0.1		
	1.0	1 636 715.0	0.1		
	192.0	65 583 388.0	0.3		
	140.0	42 413 512.0	0.3		
	98.0	179 344 312.0	0.1		
	108.0	36 600 283.0	0.3		
	1666.0	243 565 650.0	0.7		
	4.0	1 242 390.0	0.3		
	0.0	70 524.0	0.0		
	36.0	18 857 776.0	0.2		
*Median typhoid fever incidence rate per 100 000 p-years (IQR)*			0.3 (0.1, 0.4)		
**Super Region 2**					
Europe, Central [39]	0.0	7 718 750.0	0.0		
	0.0	41 386 940.0	0.0		
	5.0	40 219 115.0	0.0		
	5.0	38 157 055.0	0.0		
	17.0	43 108 829.0	0.0		
	4.0	21 596 069.0	0.0		
	22.0	8 056 366.0	0.3		
Europe, Eastern [39]	6.0	5 368 443.0	0.1		
	1.0	6 846 789.0	0.0		
	2.0	10 119 513.0	0.0		
Asia, Central	–	–	–		
*Median typhoid fever incidence rate per 100 000 p-years (IQR)*			<0.1 (0, <0.1)		
**Super Region 3**					
Sub-Saharan Africa, Southern [14]	173.0	20 459.3	845.6		
Sub-Saharan Africa, Central	–	–	–		
Sub-Saharan Africa, West	–	–	–		
Sub-Saharan Africa, East [40]	794.0	131 550.1	603.6		
			**<5 y**	**≥5 y**	**All ages**
*Median typhoid fever incidence rate per 100 000 p-years (IQR)*			2552.3^†^	366.6^†^	724.6 (603.6, 845.6)
**Super Region 4**					
North Africa/Middle East [15-17]	60.0	124 590.0	48.2		
	28.0	221 333.3	12.7		
	547.9	933 333.3	58.7		
*Median typhoid fever incidence rate per 100 000 p-years (IQR)*			48.2 (12.7, 58.7)		
**Super Region 5**					
Asia, South [18-27]	49.0	12 407.0	394.9		
	63.0	6454.0	976.1		
	58.0	27 670.5	209.6		
	129.0	31 727.0	406.6		
	78.0	19 161.0	407.1		
	122.0	56 946.0	214.2		
	189.0	41 845.0	451.7		
	32.0	4887.5	654.7		
	60.0	15 219.0	394.2		
	78.0	57 075.0	136.7		
	49.0	29 170.0	168.0		
	80.0	56 946.0	140.5		
	96.0	37 608.0	255.3		
			**<5 y**	**≥5 y**	**All ages**
*Median typhoid fever incidence rate per 100 000 p-years (IQR)*			2104.1^†^	187.0^†^	394.2 (209.6, 407.1)
**Super Region 6**					
Asia, East [20,22,23,28]	23.0	104 474.7	22.0		
	5.0	17 124.0	29.2		
	15.0	97 928.0	15.3		
	15.0	98 376.0	15.2		
Asia, South East [20,22,23,29-32]	56.0	28 329.0	197.7		
	56.0	13 538.3	413.6		
	208.0	25 670.0	810.3		
	58.0	32 164.0	180.3		
	16.0	66 165.0	24.2		
	131.0	160 261.0	81.7		
	18.0	844 55.0	21.3		
	132.0	160 257.0	82.4		
	59.0	262 699.0	22.5		
*Median typhoid fever incidence rate per 100 000 p-years (IQR)*			29.2 (22.0, 180.3)		
**Super Region 7**					
Caribbean	–	–	–		
Latin America, Andean	–	–	–		
Latin America, Central	–	–	–		
Latin America, Tropical	–	–	–		
Oceania [33,34]	275.0	1 672 000.0	16.4		
	275.0	979 781.0	28.1		
*Median typhoid fever incidence rate per 100 000 p-years (IQR)*			22.3 (16.4, 28.1)		

IQR – interquartile range

*Super Regions as defined by the Global Burden of Disease Project (Super Region 1: Australasia, Southern Latin America, High Income North America, High Income Asia Pacific; Super Region 2: Western Europe, Eastern Europe, Central Europe, Central Asia; Super Region 3: Southern Sub-Saharan Africa, Central Sub-Saharan Africa, West Sub-Saharan Africa, East Sub-Saharan Africa; Super Region 4: Northern Africa/Middle East; Super Region 5: South Asia; Super Region 6: East Asia, South East Asia; Super Region 7: Caribbean, Andean Latin America, Central Latin America, Tropical Latin America, Oceania) [7].

^†^Derived from the following data: estimated annual number of typhoid fever episodes, median proportion of cases <5 and ≥5 y of age and age-specific population estimates.

**Table 2:** Input data for paratyphoid fever incidence rates and summarized median incidence and mortality rates by Super Region*

Super Region	Paratyphoid fever cases	Person-years	Incidence (episodes/100 000 p-years)	Super Region incidence (episodes/100 000 p-years)	Super Region mortality (deaths/100 000 p-years)
				Median (IQR)	Median (IQR)
**Super Region 1**				**8.0 (0.3, 20.6)**	**<0.1 (0, 0.1)**
Australia/New Zealand [36]	471.0	77 318 800	0.6		
Latin America, Southern [11,12]	21.0	136 525	15.4		
	17.0	65 718	25.9		
North America, High Income	–	–	–		
Asia Pacific, High Income [38]	219.0	1 021 033 000	0.0		
Europe, Western	–	–	–		
**Super Region 2†**				**8.0 (0.3, 20.6)**	**<0.1 (0, 0.1)**
Europe, Central	–	–	–		
Europe, Eastern	–	–	–		
Asia, Central	–	–	–		
**Super Region 3†**				**77.4 (42.0, 130.3)**	**0.4 (0.2, 0.7)**
Sub-Saharan Africa, Southern	–	–	–		
Sub-Saharan Africa, Central	–	–	–		
Sub-Saharan Africa, West	–	–	–		
Sub-Saharan Africa, East	–	–	–		
**Super Region 4**				**0.8 (N/A)**	**<0.1 (N/A)**
North Africa/Middle East [17]	7.0	933 333	0.8		
**Super Region 5**				**77.4 (42.0, 130.3)**	**0.4 (0.2, 0.7)**
Asia, South [21,23,25-27]	38.0	19 161	198.3		
	11.0	15 219	72.3		
	24.0	57 075	42.0		
	7.0	29 170	24.0		
	47.0	56 946	82.5		
	49.0	37 608	130.3		
**Super Region 6**				**17.9 (8.8, 27.4)**	**0.1 (0, 0.1)**
Asia, East [23,28]	5.0	104 475	4.8		
	27.0	98 376	27.4		
Asia, South East [23,29,31,32]	3.0	1 353 830	0.2		
	48.0	25 670	187.0		
	22.0	160 257	13.7		
	23.0	262 699	8.8		
**Super Region 7†**				**17.9 (8.8, 27.4)**	**0.1 (0, 0.1)**
Caribbean	–	–	–		
Latin America, Andean	–	–	–		
Latin America, Central	–	–	–		
Latin America, Tropical	–	–	–		
Oceania	–	–	–		

p-years – person-years, IQR – interquartile range, N/A – not applicable

*Super Regions as defined by the Global Burden of Disease Project (Super Region 1: Australasia, Southern Latin America, High Income North America, High Income Asia Pacific; Super Region 2: Western Europe, Eastern Europe, Central Europe, Central Asia; Super Region 3: Southern Sub-Saharan Africa, Central Sub-Saharan Africa, West Sub-Saharan Africa, East Sub-Saharan Africa; Super Region 4: Northern Africa/Middle East; Super Region 5: South Asia; Super Region 6: East Asia, South East Asia; Super Region 7: Caribbean, Andean Latin America, Central Latin America, Tropical Latin America, Oceania) [7].

^†^Extrapolation used to derive Super Region incidence estimate.

Input data for typhoid and paratyphoid fever were grouped into 7 Super Regions and median incidence rates and interquartile ranges are presented in **Tables 1** and **2**, respectively. Paratyphoid fever incidence estimates were extrapolated between Super Regions on the basis of typhoid fever burden estimates. No paratyphoid fever data were available for Super Region 2 (Central Europe, Eastern Europe, Central Asia), Super Region 3 (Southern Sub-Saharan Africa, Central Sub-Saharan Africa, West Sub-Saharan Africa, East Sub-Saharan Africa), and Super Region 7 (Caribbean, Andean Latin America, Central Latin America, Tropical Latin America, Oceania). Extrapolations were made from Super Region 1 (Australia/New Zealand, Southern Latin America, High Income North America, High Income Asia Pacific, Western Europe) to Super Region 2; Super Region 5 (South Asia) to Super Region 3; and Super Region 6 (East Asia and South East Asia) to Super Region 7.

Twenty-two (88%) of the 25 eligible typhoid fever incidence studies contained age-specific typhoid fever data for children <5 years and persons ≥5 and older (**Table 3**). Age-specific data were available for 6 (29%) of 21 regions representing 5 of 7 Super Regions. All data came from low- and middle-income countries. The median proportion of typhoid fever episodes among children <5 years was 57.7%, and among persons ≥5 years, 42.3%. For Super Regions 3 and 5, the median proportion of typhoid fever cases among each age strata was used to calculate regional estimates of annual number of cases and incidence rates for each age group (**Table 4**).

**Table 3:** An overview of studies with age-specific typhoid fever incidence rates by Super Regions

Super Region*	<5 y				≥5 y			
	Typhoid fever cases	Person-years	Incidence (cases/100 000 p-years)	Proportion of overall (%)	Typhoid fever cases	Person-years	Incidence (cases/100 000 p-years)	Proportion of overall (%)
Latin America, Southern [11-13]	–	–	–	–	68.0	65 718.0	103.5	N/A
	–	–	–	–	28.0	30 906.0	91.0	N/A
Sub-Saharan Africa, East [40]	240.9	23 167.0	1039.9	30.3	553.0	108 383.1	510.3	69.7
North Africa / Middle East [15-17]	9.2	157 631.7	5.8	7.6	545.8	775 114.2	70.4	92.4
Asia, South [18-27]	26.0	1393.0	1870.0	89.9	23.0	11 014.0	210.0	10.1
	28.0	1027.0	2726.4	80.9	35.0	5427.0	644.9	19.1
	11.0	4061.0	270.9	56.4	111.0	52 885.0	209.8	43.6
	58.0	10 118.0	573.2	58.1	131.0	31 727.0	412.9	41.9
	13.0	15 545.5	83.6	24.0	36.0	13 625.0	264.2	76.0
	27.0	2089.6	1292.0	86.6	69.0	34 543.9	199.7	13.4
Asia, East [20,22,23,28]	0.0	489.25	0.0	0.0	23.0	103 985.4	22.1	100.0
	–	–	–	–	15.0	97 928.0	15.3	N/A
Asia, South East [20,22,23,29-32]	26.0	1989.3	1307.0	62.9	182.0	23 658.9	769.3	37.1
	14.0	12 924.0	108.3	57.7	117.0	147 337.0	79.4	42.3
	–	–	–	–	18.0	84 455.0	21.3	N/A
*Median proportion of cases split by <5 y and 5 y of age and older for low and middle income countries*				57.7				42.3^†^

p-years – person-years, IQR – interquartile range, N/A – not applicable, y – years

*Super Regions as defined by the Global Burden of Disease Project (Super Region 1: Australasia, Southern Latin America, High Income North America, High Income Asia Pacific; Super Region 2: Western Europe, Eastern Europe, Central Europe, Central Asia; Super Region 3: Southern Sub-Saharan Africa, Central Sub-Saharan Africa, West Sub-Saharan Africa, East Sub-Saharan Africa; Super Region 4: Northern Africa/Middle East; Super Region 5: South Asia; Super Region 6: East Asia, South East Asia; Super Region 7: Caribbean, Andean Latin America, Central Latin America, Tropical Latin America, Oceania) [7].

^†^Derived from the median proportion of overall cases attributable to children under 5 y of age.

**Table 4:** Annual number of typhoid fever episodes, 2010 by Super Region*

Super Region	2010		
**Super Region 1**	**All ages**		
Population [41]	1 019 736 630.0		
Median typhoid fever incidence rate per 100 000 p-years (IQR)	0.3 (0.1, 0.4)		
Annual number of typhoid fever episodes (IQR)	3059.2 (1019.7, 4078.9)		
**Super Region 2**	**All ages**		
Population [41]	406 303 917.1		
Median typhoid fever incidence rate per 100 000 p-years (IQR)	<0.1 (0, <0.1)		
Annual number of typhoid fever episodes (IQR)	406.3 (0, 406.3)		
**Super Region 3**	**<5 y**	**≥5years**	**All ages**
Population [41]	140 250 136.0	715 910 880.0	856 161 016.0
Median typhoid fever incidence rate per 100 000 p-years (IQR)	2552.3	366.6	724.6 (603.6, 845.6)
Annual number of typhoid fever episodes (IQR)	3 579 559.6**^†^**	2 624 183.2**^†^**	6 203 742.7 (5 167 787.9, 7 239 697.6)
**Super Region 4**	**All ages**		
Population [41]	4 454 87 756.0		
Median typhoid fever incidence rate / 100 000 p-years (IQR)	48.2 (12.7, 58.7)		
Annual number of typhoid fever episodes (IQR)	214 725.1 (56 576.9, 261 501.3)		
**Super Region 5**	**<5 y**	**≥5 y**	**All ages**
Population [41]	174 016 500.0	1 435 769 400.0	1 609 785 900.0
Median typhoid fever incidence rate per 100 000 p-years (IQR)	2104.1	187.0	394.2 (209.6, 407.1)
Annual number of typhoid fever episodes (IQR)	3 661 512.8**^†^**	2 684 263.2**^†^**	6 345 776.0 (3 374 111.3, 6 553 438.4)
**Super Region 6**	**All ages**		
Population [41]	2 016 815 598.0		
Median typhoid fever incidence rate per 100 000 p-years (IQR)	29.2 (22.0, 180.3)		
Annual number of typhoid fever episodes (IQR)	588 910.2 (443 699.4, 3 636 318.5)		
**Super Region 7**	**All ages**		
Population [41]	528 026 317.0		
Median typhoid fever incidence rate per 100 000 p-years (IQR)	22.3 (16.4, 28.1)		
Annual number of typhoid fever episodes (IQR)	117 749.9 (865 96.3, 148 375.4)		
**Global Total: annual number of typhoid fever episodes (crude (IQR) / adjusted (IQR) ‡)**	**13 474 369.4 (9 129 791.5, 17 843 816.4) / 26 948 738.8 (18 259 583.0, 35 687 632.8)**		

p-years – person-years, IQR – interquartile range

*Super Regions as defined by the Global Burden of Disease Project (Super Region 1: Australasia, Southern Latin America, High Income North America, High Income Asia Pacific; Super Region 2: Western Europe, Eastern Europe, Central Europe, Central Asia; Super Region 3: Southern Sub-Saharan Africa, Central Sub-Saharan Africa, West Sub-Saharan Africa, East Sub-Saharan Africa; Super Region 4: Northern Africa/Middle East; Super Region 5: South Asia; Super Region 6: East Asia, South East Asia; Super Region 7: Caribbean, Andean Latin America, Central Latin America, Tropical Latin America, Oceania) [7].

^†^Derived from median proportion of cases <5 and ≥5 y of age (Table 5).

‡Adjusted to account for low sensitivity of blood culture typhoid test.

The median typhoid fever incidence rate for each Super Region applied to the 2010 population estimates generates a crude global estimate of 13474369 typhoid fever episodes each year (**Table 4**). After adjusting for the low sensitivity of the blood culture typhoid test we estimate typhoid fever incidence to be 26948739 episodes annually.

There is little data to describe typhoid or paratyphoid fever case-fatality rates. In the most recent study on the global typhoid fever burden, Crump et al. assumed a case-fatality rate of 1% for typhoid fever based on hospital-based data, expert opinion, and mortality rates documented by advanced national surveillance systems [1]. Given we found no new data to suggest an improvement in typhoid fever case fatality rates, we also used this figure to estimate the total number of annual deaths and to derive mortality estimates, which are presented in **Table 5**. Past studies on the global paratyphoid fever burden have not reported mortality estimates. Our study assumed a case-fatality rate of 0.5% given that paratyphoid fever is generally less severe than typhoid fever [42]. Mortality estimates for paratyphoid fever are presented in **Table 2**.

**Table 5:** Summarized median typhoid fever mortality rates by Super Region*

Super Region	Region	Super Region mortality (deaths/ 100 000 p-years) [1]
		Median (IQR)
**Super Region 1**	Australia/New Zealand [35,36]	<0.1 (0, <0.1)
	Latin America, Southern [11-13]	
	North America, High Income [37]	
	Asia Pacific, High Income [38]	
	Europe, Western [39]	
**Super Region 2**	Europe, Central [39]	<0.1 (0, <0.1)
	Europe, Eastern [39]	
	Asia, Central	
**Super Region 3**	Sub-Saharan Africa, Southern [14]	7.2 (6.0, 8.5)
	Sub-Saharan Africa, Central	
	Sub-Saharan Africa, West	
	Sub-Saharan Africa, East [40]	
**Super Region 4**	North Africa/Middle East [15-17]	0.5 (0.1, 0.6)
**Super Region 5**	Asia, South [18-27]	3.9 (2.1, 4.1)
**Super Region 6**	Asia, East[20,22,23,28]	0.3 (0.2, 1.8)
	Asia, South East [20,22,23,29-32]	
**Super Region 7**	Caribbean	0.2 (0.2, 0.3)
	Latin America, Andean	
	Latin America, Central	
	Latin America, Tropical	
	Oceania [33,34]	

p-years – person-years, IQR – interquartile range

*Super Regions as defined by the Global Burden of Disease Project (Super Region 1: Australasia, Southern Latin America, High Income North America, High Income Asia Pacific; Super Region 2: Western Europe, Eastern Europe, Central Europe, Central Asia; Super Region 3: Southern Sub-Saharan Africa, Central Sub-Saharan Africa, West Sub-Saharan Africa, East Sub-Saharan Africa; Super Region 4: Northern Africa/Middle East; Super Region 5: South Asia; Super Region 6: East Asia, South East Asia; Super Region 7: Caribbean, Andean Latin America, Central Latin America, Tropical Latin America, Oceania) [7].

## DISCUSSION

Our results suggest that in 2010, there were an estimated 13.5 million typhoid fever episodes globally. This estimate is comparable to the 2000 crude estimate of 10.8 million episodes published by Crump et al [1]. We sought to update the previous estimate and in doing so, found a number of more recently published studies with higher incidence rates than those reported in older studies that influenced our final estimate. We used slightly different inclusion and exclusion criteria and applied slightly different methods for estimating incidence globally from the previous systematic review, which all contributed to the observed differences. However, given that the world’s population has grown by 10% in the last 10 years, our revised estimate, compared to the previously published 2000 estimate, is well within a plausible margin of error.

Quantity of source data remains a major limitation for estimating the global burden of typhoid and paratyphoid fever. While additional data on paratyphoid fever is needed across all regions, typhoid fever estimates are limited by the scarcity of reliable incidence data from many of the developing regions in particular. Lacking surveillance systems or eligible population-based studies, typhoid fever incidence data were unavailable for 7 (33%) regions including: Central Asia, Central Sub-Saharan Africa, West Sub-Saharan Africa, Caribbean, Andean Latin America, Central Latin America, and Tropical Latin America. Furthermore, incidence estimates for several regions were based on few studies. Of note, we identified only 5 eligible studies conducted in Africa. As a result, our estimate for Super Region 3 – representing all of sub-Saharan Africa – was based on only two studies conducted in South Africa and Kenya [14,40]. Similarly, North Africa/Middle East estimates relied on only 3 studies that were carried out in Egypt [15-17]. Outside of Africa, there is also limited data available for Super Region 7. Only 2 studies from Fiji and Tonga were used to estimate the burden of disease for this region and both reported the results of pilot surveillance systems, thus there exists considerable uncertainty related to this approximation [33,34]. Additional population-based surveillance studies must be carried out in Africa and other developing regions to develop a more accurate understanding of the global typhoid fever burden.

We restricted our analysis to studies and surveillance systems that used blood culture as the criteria for diagnosis. Although typhoid and paratyphoid fever are most commonly diagnosed using this method, it is only 50% sensitive. Factors that influence test sensitivity include antimicrobial use, the volume of blood collected, and the timing of blood collection [8,10,43]. These important limitations introduce a bias toward underestimation. In contrast, the inclusion of vaccine studies as a source for incidence data promotes a bias toward overestimation as sites are generally selected for having high incidence rates due to sample size considerations.

Typhoid and paratyphoid fever are major public health problems, especially in the developing world. Our study reports a revised estimate of the global burden of these diseases based on new data available from recent population-based studies and broader coverage of surveillance systems. In total, we identified 49 sources of new data that have become available since the 2000 estimate published by Crump et al. in 2004 [1]: 15 population-based studies, 30 national-surveillance systems, and 4 partially representative surveillance systems. Collectively, these sources provide estimates of overall typhoid fever incidence rates from 14 (67%) of the 21 regions across 5 Super Regions.

Although our understanding of the global burden of these diseases has improved with more recent data, both enteric fevers remain poorly quantified. Critical gaps in our understanding persist, as the burden remains largely unknown in many of the regions. Appreciable gains would be made by: a) developing improved diagnostic methods; b) implementing surveillance systems; and c) carrying out additional population-based research, particularly in sub-Saharan Africa and other developing countries. Recent studies have shown that paratyphoid fever accounts for an increasing proportion of enteric fever in several regions [19,23,44-47]. If this trend continues, important challenges can be anticipated in the absence of an effective vaccine for this disease. In addition, multi-drug resistant *S. typhi* and *S. paratyphi* organisms may continue to increase in prevalence and could certainly hamper efforts to reduce related morbidities. An accurate epidemiological profile of the global burden of typhoid and paratyphoid fever is important to developing effective disease prevention and control strategies.
